# CD3ε^+^ Cells in Pigs With Severe Combined Immunodeficiency Due to Defects in *ARTEMIS*

**DOI:** 10.3389/fimmu.2020.00510

**Published:** 2020-03-31

**Authors:** Adeline N. Boettcher, A. Giselle Cino-Ozuna, Yash Solanki, Jayne E. Wiarda, Ellie Putz, Jeana L. Owens, Sara A. Crane, Amanda P. Ahrens, Crystal L. Loving, Joan. E. Cunnick, Raymond R. R. Rowland, Sara E. Charley, Jack C. M. Dekkers, Christopher K. Tuggle

**Affiliations:** ^1^Department of Animal Science, Iowa State University, Ames, IA, United States; ^2^Veterinary Diagnostic Laboratory, Kansas State University, Manhattan, KS, United States; ^3^Food Safety and Enteric Pathogen Unit, National Animal Disease Center, Agricultural Research Service, U.S. Department of Agriculture, Ames, IA, United States; ^4^Immunobiology Graduate Program, College of Veterinary Medicine, Iowa State University, Ames, IA, United States; ^5^Agricultural Research Service Participation Program, Oak Ridge Institute for Science and Education, Oak Ridge, TN, United States; ^6^Laboratory Animal Research, Iowa State University, Ames, IA, United States; ^7^Diagnostic Medicine and Pathobiology Department, Kansas State University, Manhattan, KS, United States

**Keywords:** SCID, severe combined immunodeficiency, artemis, T cell, swine

## Abstract

Severe combined immunodeficiency (SCID) is described as the lack of functional T and B cells. In some cases, mutant genes encoding proteins involved in the process of VDJ recombination retain partial activity and are classified as hypomorphs. Hypomorphic activity in the products from these genes can function in the development of T and B cells and is referred to as a leaky phenotype in patients and animals diagnosed with SCID. We previously described two natural, single nucleotide variants in *ARTEMIS* (*DCLR1EC*) in a line of Yorkshire pigs that resulted in SCID. One allele contains a splice site mutation within intron 8 of the *ARTEMIS* gene (*ART16*), while the other mutation is within exon 10 that results in a premature stop codon (*ART12*). While initially characterized as SCID and lacking normal levels of circulating lymphoid cells, low levels of CD3ε^+^ cells can be detected in most SCID animals. Upon further assessment, we found that *ART16/16*, and *ART12/12* SCID pigs had abnormally small populations of CD3ε^+^ cells, but not CD79α^+^ cells, in circulation and lymph nodes. Newborn pigs (0 days of age) had CD3ε^+^ cells within lymph nodes prior to any environmental exposure. CD3ε^+^ cells in SCID pigs appeared to have a skewed CD4α^+^CD8α^+^CD8β^−^ T helper memory phenotype. Additionally, in some pigs, rearranged VDJ joints were detected in lymph node cells as probed by PCR amplification of TCRδ V5 and J1 genomic loci, as well as TCRβ V20 and J1.1, providing molecular evidence of residual Artemis activity. We additionally confirmed that TCRα and TCRδ constant region transcripts were expressed in the thymic and lymph node tissues of SCID pigs; although the expression pattern was abnormal compared to carrier animals. The leaky phenotype is important to characterize, as SCID pigs are an important tool for biomedical research and this additional phenotype may need to be considered. The pig model also provides a relevant model for hypomorphic human SCID patients.

## Introduction

Artemis is involved in DNA repair and is critical for proper T and B cell receptor gene arrangement and subsequent development of naïve lymphocytes. Mutations in Artemis results in severe combined immunodeficiency (SCID), characterized by the lack of circulating T and B cells (1). We previously described two natural mutations within the *ARTEMIS* gene in two separate alleles (termed *ART16* and *ART12*) in a line of Yorkshire pigs, which leads to SCID in a homozygous or heterozygous state (2). The *ART16* allele contains a splice site mutation within intron 8 of *ARTEMIS* and all transcripts in homozygous *ART16/16* fibroblasts are missing exon 8. The *ART12* allele contains a nonsense mutation in exon 10 that leads to a premature stop codon and many of the transcripts in *ART12/12* fibroblasts are missing large portions of all exons (2). Upon the discovery of the *ART* SCID pigs, bone marrow transplantation (BMT) was performed to reconstitute the animal immune system and to further study the animals. Among the SCID pigs that underwent BMT, two *ART16/16* pigs developed host derived CD3ε^+^ T-cell lymphoma (3). This led us to hypothesize that the *ART16* allele potentially produced Artemis protein with residual activity, resulting in development of T cells. Hypomorphic mutations are reported in *RAG* (4–6) and *ARTEMIS* (7–10) in human patients, and can lead to complications such as cancer (8, 9) and Omenn's syndrome (11). Mice have also been genetically modified with hypomorphic *ARTEMIS* mutations (12, 13). Thus, it is important to understand and characterize the CD3ε^+^ cells that developed in *ARTEMIS* mutant SCID pigs, a phenotype referred to as leaky.

We describe here a leaky CD3ε^+^ cellular phenotype in pigs with SCID due to mutated *ART*. Tissues and blood were collected from pigs across the three different SCID genotypes (*ART12/12, ART12/16*, and *ART16/16*) at various ages, and pigs from all genotypes had some level of CD3ε^+^ cells. We detected CD79α^+^ cells in the lymph node in only one *ART16/16* pig out of 25 total SCID pigs tested. We performed PCR on SCID pig lymph node DNA and found evidence of VDJ recombination in both the TCRδ and TCRβ loci. We additionally confirmed the expression of TCRα and TCRδ constant region transcripts utilizing a two-color RNA *in-situ* hybridization technique to observe αβ and γδ T cells, respectively. Taken together, occurrence of VDJ recombination within the TCR loci and expression of TCR transcripts in lymphoid tissues show that CD3ε^+^ cells are capable of developing in SCID pigs with mutations in *ART*. Documentation of this leaky CD3ε^+^ cellular phenotype is important as this animal model is further developed for biomedical research.

## Materials and Methods

### Ethics Statement

All animal procedures and protocols were approved by Iowa State University's Institutional Animal Care and Use Committee.

### Generation of Piglets and Rearing

SCID pigs were derived as previously described (2) and housed in either clean conventional rooms or in biocontainment facilities (14). All piglets were either handfed 250 mL colostrum or naturally suckled on the sow after birth. All pigs, genotypes, housing conditions, age, CD3ε result summaries, analysis methods, and figure references are in Table 1. There was no overlap in pigs between this study and those described in Waide et al. (2).

**Table 1:** Overview of SCID pigs, genotypes, and description of CD3ε^+^ and CD79α^+^ cells in blood or lymph nodes.

**Pig #**	***ART* genotype**	**Housing**	**Age**	**Immunophenotype**	**Analysis**	**Figure Reference**
1	12/+	C	7 W	Control animal; CD3ε^+^ cells in PB	F	1
2	12/16	C	7 W	CD3ε^+^ CD16^−^ cells in PB	F	1
3	16/16	C	7 W	CD3ε^+^ CD16^−^ cells in PB	F	1
4	16/16	B	4 M	Low levels of CD3ε^+^ cells in PB; thymic tissue with CD3ε^+^ cells present; LN with CD3ε^+^ cells present	F, IHC	2
5	16/16	B	4 M	Low levels of CD3ε^+^ cells in PB	F	2
6	12/12	B	3 M	CD3ε^+^ cells present in PB; TCRδ rearrangement	F, PCR	2,5
7	12/16	B	3 M	CD3ε^+^ cells present in PB	F	2
8	12/+	B	4 M	Control animal; CD3ε^+^ and CD79α^+^ cells in PB	F	2
9	16/16	N/A	0 D	No CD3ε^+^ or CD79α^+^ cells in LN	IHC	3
10	16/16	N/A	0 D	CD3ε^+^ and CD79α^+^ cells present in LN	IHC	3
11	12/16	N/A	0 D	CD3ε^+^ cells present, and no CD79α^+^ cells present in LN	IHC	3
12	12/16	N/A	0 D	Punctate CD3ε^+^ cells present, and no CD79α^+^ cells present in LN	IHC	3
13	12/12	N/A	0 D	Punctate CD3ε^+^ cells present, and no CD79α^+^ cells present in LN	IHC	3
14	12/12	N/A	0 D	Punctate CD3ε^+^ cells present, and no CD79α^+^ cells present in LN	IHC	3
15	12/+	N/A	0 D	Control animal; CD3ε^+^ and CD79α^+^ cells present in LN	IHC	3
16	16/16	B/C	6 M	CD3ε^+^ cells present in PB; CD3ε^+^ cells present, and no CD79α^+^ cells present in LN	F, IHC	4
17	16/16	C	5.5 M	CD3ε^+^ cells present, and no CD79α^+^ cells present in LN. SWC6^+^ cells present in LN; TCRβ recombination	F, IHC, PCR	4,5
18	16/+	C	6 W	Control animal; TCRδ or TCRβ rearrangement	PCR	5
19	16/16	C	6 W	No TCRδ or TCRβ rearrangement	PCR	5
20	16/16	C	6 W	TCRδ and TCRβ rearrangement	PCR	5
21	16/16	C	6 W	TCRδ and TCRβ rearrangement	PCR	5
22	12/16	C	6 W	TCRβ rearrangement	PCR	5
23	12/16	B	11 W	TCRδ rearrangement	PCR	5
24	12/16	B	4 W	TCRδ and TCRβ rearrangement	PCR	5
25	12/12	B	10 W	No TCRδ or TCRβ rearrangement	PCR	5
26	12/+	B	7 W	Control animal; αβ and γδ T cells in thymus and lymph node	ISH	6
27	12/6	B	7 W	αβ and γδ T cells in thymus and lymph node	ISH	6
28	12/16	B	7 W	αβ and γδ T cells in thymus and lymph node	ISH	6
29	12/16	B	9 W	αβ and γδ T cells in thymus and lymph node	ISH	6
30	12/16	B	9 W	αβ and γδ T cells in thymus and lymph node	ISH	6

*C, Conventional housing; B, Biocontainment; PB, peripheral blood; LN, lymph node; F, Flow cytometry; IHC, immunohistochemistry; PCR, polymerase chain reaction; TCR δ, Recombination between V5 and J1; TCR β, Recombination between V20 and J1.2; ISH, RNA in-situ hybridization*.

### Flow Cytometry of PBMCs and MNCs

PBMCs were isolated as previously described (15). To collect lymph node mononuclear cells (MNCs), lymph node tissue was dissected and placed in HBSS with 10 μg/mL gentamicin. In the lab, tissues were minced in a digestion solution of HBSS with 300 mg/L of collagenase, 3% FBS, and 2 mM HEPES (referred to herein as d-HBSS). The tissue and d-HBSS mixture was incubated for 1 h at 37°C with vortexing every 15 min. The suspension was passed through a 100 μm cell strainer to isolate MNCs and was washed once in d-HBSS. Isolated PBMCs and MNCs were counted by flow cytometry using a BD cell viability counting kit.

PBMCs and MNCs were stained with the following antibodies: anti-pig CD16 (G7, Bio-Rad) with a goat anti-mouse IgG1 secondary antibody, anti-pig CD3ε (BB23-8E6-8C8), anti-human CD79α (HM7), anti-pig CD8β (PPT23), anti-pig CD8α (76-12-4), anti-pig CD4α (74-12-4), and anti-pig SWC6 (MAC320). SWC6 is an uncharacterized membrane protein and the antibody (MAC320) identifies a population of γδ TCR^+^ cells in the blood (16, 17). Cells were fixed in 2% PBS formaldehyde and data was acquired with a FACS Canto II and subsequently analyzed using FlowJo (Tree Star).

### Immunohistochemistry of Collected Lymphoid Tissues

Pigs were euthanized via intravenous injection of pentobarbital sodium (Fatal Plus). Lymph node and thymic tissues were collected and placed into 10% buffered formalin for 24 hours. Tissues were then moved to 70% ethanol until processing. Immunohistochemical staining for T and B lymphocyte markers was performed in paraffin-embedded tissue thin sections at Kansas State Veterinary Diagnostic Laboratory (KSVDL). Briefly, deparaffinized slide-mounted thin sections were pre-treated for 5 min with a peroxide block, followed by incubation with primary antibody. Primary antibodies included mouse anti-CD3ε (clone LN10, Leica Biosystems) and mouse anti-CD79α (clone HM57, Abcam). Primary antibodies were incubated for 25 min with PowerVision Poly-HRP anti-mouse IgG at room temperature, with DAB chromagen, and counterstained with hematoxylin.

### PCR of Lymph Node DNA for TCRβ and TCRγ V and J Rearrangement

PCR analysis was performed on DNA isolated from various lymph node tissues (thoracic, inguinal, and subscapular) collected from SCID and carrier pigs. We used a protocol similar to Suzuki et al. (18) to assess TCR rearrangements, with the addition of an in-house designed TCRβ J1.2 reverse primer (see below)(19). Zymo Research Genomic-DNA™ Tissue Miniprep Kit was used to extract genomic DNA from the tissue. A 40 μl PCR reaction volume was used with 160 ng of gDNA was used as a template, and amplification was performed with Promega GoTaq® Green Master Mix. TCRβ recombination was assessed using primers designed for TCRβV20 (5′ GATGTCATGGACATCATTTGCCATC 3′) (18) and TCRβJ1.2 (5′ GGGCCGAAGTTATAGTCATA 3′). TCRδ recombination was assessed using primers designed for TCRδV5 (5′ TTCAGACACGTGACCTTCAG 3′) (18) and TCRδJ1 (5′ GTTCCACAACCAGCTGAGTC 3′) (18). Germline sequence of TCRβV20 was amplified as a positive control using the TCRβV20 primer listed above with a reverse primer (5′ GCTGAGATTCTGGGATTCAC 3′). All samples used the same thermal cycling parameters of 94°C for 10 min, followed by 39 cycles of 95°C for 30 s, 62°C for 30 s, 72°C for 1 min 30 s, followed by a final step of 72°C for 10 min.

### *In-situ* Hybridization of TRDC and TRAC Constant Region Transcripts in Lymphoid Tissues

Fixed tissues were prepared as described above. Chromogenic 2-color RNA *in-situ* hybridization was performed using the RNAscope 2.5 HD Duplex kit (Advanced Cell Diagnostics). Custom probes complementary to porcine TRDC (ACD catalog no. 553141) and TRAC (ACD catalog no. 565291) mRNA were created to detect γδ and αβ T cells, respectively. RNAscope staining was carried out according to manufacturer's instructions with the following adjustments: (1) target retrieval was carried out by incubating slides in 1X Target Retrieval Solution (ACD) at 95°C for 15 min in a pressurized Decloaking Chamber NxGen (BioCare) and (2) Protease Plus (ACD) was applied to each tissue section for 15 min at 40°C.

## Results

### Peripheral CD16^−^ CD3ε^+^ Cells Detected in SCID Pigs Raised in Conventional Housing

**Figure 1:**
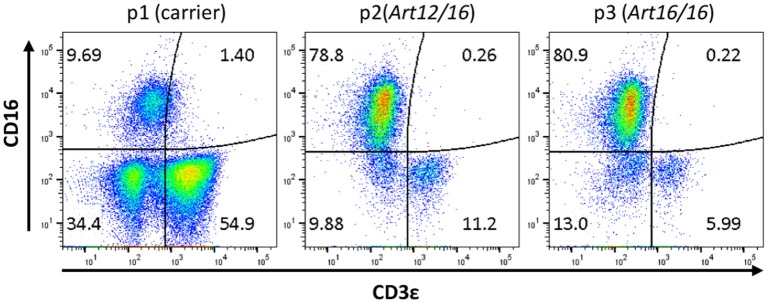
SCID pigs have CD3ε^+^ CD16^−^ cells in circulation. PBMCs from a carrier (p1) and SCID pigs (p2 [ART12/16] and p3 [ART16/16]) housed in conventional clean rooms were stained to determine CD16 and CD3ε expression.

The initial detection of circulating CD3ε^+^ cells in SCID pigs occurred during a routine engraftment check on an *ART*^−/−^ SCID pig that had previously received a bone marrow transplantation. During the engraftment check, peripheral blood mononuclear cells (PBMCs) were assessed for CD3ε expression from the putative BMT, as well as a carrier (*ART*^+/−^*)* and SCID (*ART*^−/−^) pigs as control cells for the assay. Flow cytometry analysis revealed the SCID pig tested had a small population of CD3ε^+^ cells in circulation (data not shown). Blood was tested again from a carrier (Pig 1; p1), *ART12/16* (p2), and *ART16/16* (p3) littermate pigs, all of which were 7 weeks of age and raised in conventional (non-biocontainment) rooms (Figure 1). NK cells develop in *ART*^−/−^ SCID pigs and retain function *in vitro*(15); thus PBMCs from the indicated animals were labeled for CD3ε and CD16 (20) to evaluate for NKT cells. CD3ε^+^ cells in SCID pigs were detected again, confirming the earlier observation of “leaky” SCID phenotype (13). All SCID pig CD3ε^+^ populations were CD16^−^, suggesting that they were not NKT cells (Figure 1).

### SCID Pigs in Biocontainment Facilities Develop CD3ε^+^, But Not CD79α^+^ Cells

**Figure 2:**
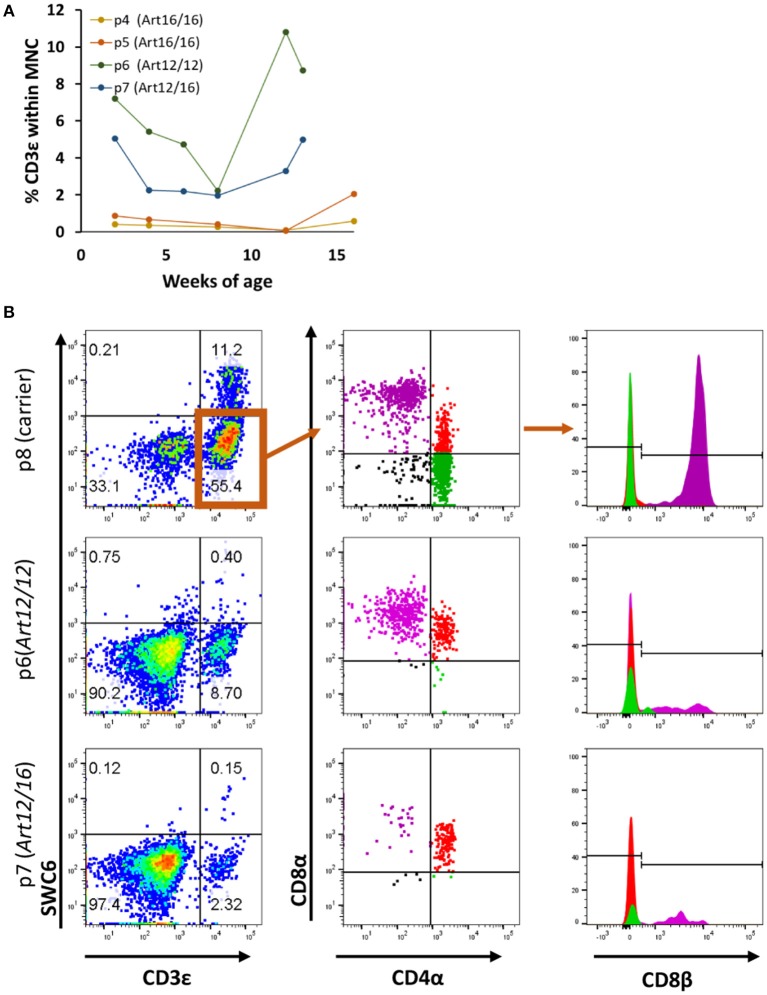
SCID pig CD3ε^+^ cells are primarily cytotoxic T and memory T cell in circulation. **(A)** Four SCID pigs (p4 [*ART16/16*], p5 [*ART16/16*], p6 [*ART12/16*], and p7 [*ART12/12*]) were monitored over a three to four-month period for circulating CD3ε^+^ cells. P6 and p7 had variable levels of CD3ε^+^ cells throughout this period. **(B)** CD3ε^+^ cells from p6 and p7 were assed for expression of SWC6, CD4α, CD8α, and CD8β. CD3ε^+^ cells within the SCID pigs were primarily CD8α^+^ CD8β^+^ CD4α^−^ and CD8α^+^ CD8β^−^ CD4α^+^.

We next investigated when CD3ε^+^ cells developed in *ART*^−/−^ SCID pigs and if raising the pigs in clean biocontainment facilities (14) would reduce the prevalence of CD3ε^+^ cellular expansion, as there would be a reduced pathogen exposure compared to standard conventional housing settings (14). PBMCs from four pigs born and raised in positive pressure biocontainment facilities (14) over a 3- to 4-month period were isolated and labeled for CD3ε and CD79α expression to assess T and B cell development. PBMC from carrier animals (*ART*^+/−^) were stained alongside SCID pig PBMC samples, and carriers consistently had 70–90% CD3ε^+^ cells and 5–15% CD79α^+^ cells within the lymphocyte population (data not shown). PBMCs from SCID pigs were first gated on total mononuclear cells (lymphocytes and myeloid cells) as opposed to lymphocytes only due to inability to distinguish between the two populations by forward scatter (FSC) and side scatter (SSC) in the blood samples. *ART16/16* animals (p4 and p5) had low to no circulating CD3ε^+^ cells, while the *ART12/12* (p6) and *ART12/16* (p7) pigs had variable frequencies of CD3ε^+^ cells (Figure 2A). Interestingly, upon necropsy at 4 months of age, p4 had thymic tissue (Supplemental Figure 1) which immunohistochemistry indicated as predominantly CD3ε^+^ cells. CD79α^+^ cells were not detected in the blood during the 3–4 month period tested (data not shown).

We next sought to determine the cellular phenotypes of the CD3ε^+^ cells in the SCID pigs. Normal swine have T helper cells (CD4^+^CD8α^−^CD8β^−^), memory T helper cells (CD4^+^CD8α^+^CDβ^−^) and cytotoxic T cells (CD4^−^CD8α^+^CD8β^+^) (20). To assess the cellular phenotypes, PBMCs were stained for SWC6, CD4α, CD8α, and CD8β. SWC6 is expressed on a majority of γδ T cells found within the blood (16, 17). PBMCs from p6 (*ART12/12*), p7 (*ART12/16*), and p8 (carrier), as a control, were analyzed for these T cell markers at 3 months of age. Both SCID animals appeared to have a very small population of CD3ε^+^ SWC6^+^ cells (Figure 2B). The CD3ε^+^ SWC6^−^ population in both p6 and p7 were primarily CD8α^+^ CD8β^+^ CD4α^−^ and CD8α^+^ CD8β^−^ CD4α^+^. T helper memory cells in swine have a CD8α^+^ CD8β^−^ CD4α^+^ cellular phenotype (21).

### CD3ε^+^ Cells in the Lymph Nodes of Neonatal SCID Pigs

**Figure 3:**
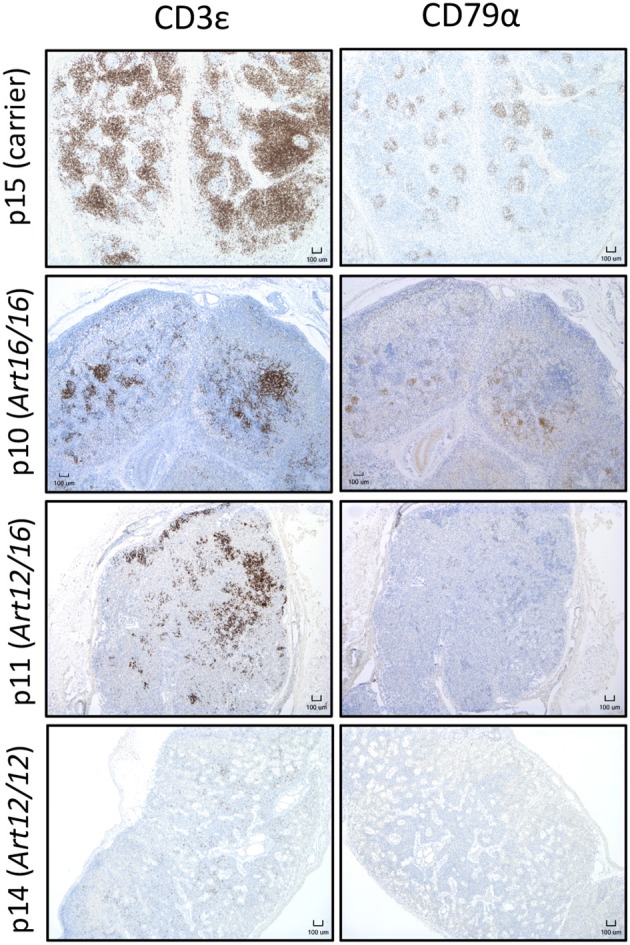
CD3ε^+^ cells are found in neonatal SCID pig lymph nodes. Lymph nodes were collected from 0 day old SCID pigs (p15 [carrier], p10 [*ART16/16*], p11 [*ART12/16*], p14 [*ART12/12*]) and were stained for CD3ε and CD79α. Indicated cells are labeled as brown.

Since we found that CD3ε^+^ cells circulate in older animals, we next asked if these cells were present in neonatal SCID pigs. Lymph nodes were collected from 0 day old (0-6 hours old and given no colostrum) SCID pigs with *ART16/16* (p9 and p10), *ART12/16* (p11 and p12), *ART12/12* (p13 and p14), and carrier (p15) genotypes and assessed for the presence of CD3ε^+^ and CD79α^+^ cells by immunohistochemistry. Two animals for each genotype were assessed; representative histology images are shown in Figure 3. Lymph nodes from SCID animals had abnormal architecture with poorly defined medulla and cortex structures. All SCID genotypes had some level of CD3ε^+^ cells present. The p13 and p14 (*ART12/12*) animal's lymph nodes had fewer CD3ε^+^ cells, which were punctate within the lymph node. CD79α^+^ cells were only found in one pig (p10, *ART16/16*), out of 25 that were tested. These results indicate that CD3ε^+^ cells, and in rare cases CD79α^+^ cells, initiate development *in utero* in *ART*^−/−^ SCID pigs.

### CD3ε^+^ Cells in 5- and 6-Month-Old SCID Pigs

**Figure 4:**
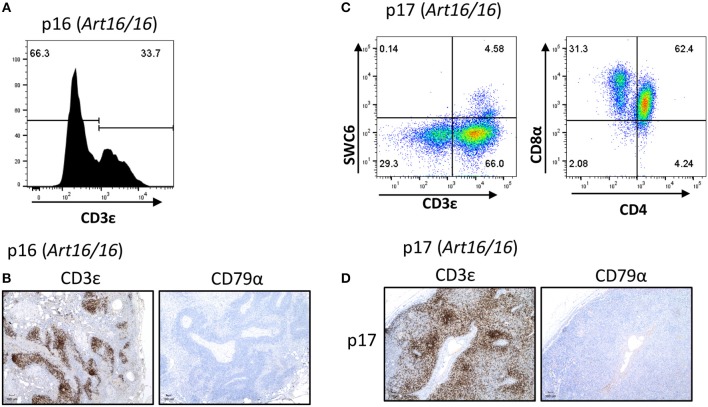
CD3ε^+^ cells in a 5- and 6-month-old SCID pigs. **(A)** PBMCs (gated on lymphocytes) were stained for CD3ε expression from a 6-month-old *ART16/16* SCID (p16). **(B)** Lymph nodes were stained for CD3ε and CD79α expression from p16. **(C)** Mononuclear cells were isolated from a popliteal lymph node from p17 and stained for CD3ε, SWC6, CD4, and CD8α. A small population of CD3ε^+^ SWC6^+^ cells were present. A majority of the CD3^+^ SWC6^−^ population were CD4α^+^ CD8α^+^. **(D)** Lymph nodes were stained for CD3 and CD79α expression from p17 [*ART16/16*].

We additionally collected lymph nodes from *ART16/16* SCID pigs that were 5.5 and 6 months of age. Pig p16 was originally raised in biocontainment facilities and was later moved to conventional housing at approximately 3 months of age. At 6 months of age the pig developed severe skin lesions and was euthanized due to failure to thrive. At the time of euthanasia, a blood sample was collected from this animal, and 33% of lymphocytes were found to be CD3ε^+^ (Figure 4A). Other T cell markers were not assessed at this time. Lymph nodes were also collected, and CD3ε^+^, but not CD79α^+^, cells were present (Figure 4B).

Another older *ART16/16* animal, p17, was also raised to 5 and a half months of age in biocontainment facilities. The pig was diagnosed with an *E. coli* bacterial infection and was euthanized due to failure to thrive. Lymph nodes were collected from this animal and were assessed by flow cytometry and histological analysis (Figures 4C,D). MNCs from a popliteal lymph node were isolated and analyzed by flow cytometry. Of gated MNCs, 66% of cells were CD3ε^+^. A small portion of the CD3ε^+^ cells were also SWC6^+^. CD3ε^+^ cells in p17 lymph nodes consisted of CD8α^+^ CD4α^−^ and CD8α^+^ CD4α^+^. Expression of CD8β was not assessed at this time. Immunohistochemistry revealed high prevalence of CD3ε^+^ cells in the lymph node. CD79α^+^ cells were not present in lymph nodes.

### Evidence of Limited TCR Rearrangement in ART^–/–^ SCID Pigs

**Figure 5:**
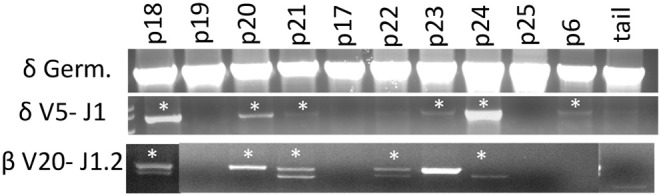
Cells with TCRδ V5-J1 and TCRβV20-J1.2 recombination are in SCID pig lymph nodes. DNA from lymph nodes were amplified with primers specific for TCRδV5 – TCRδJ1, as well as TCRβ V20- TCRβ J1.1 from a carrier and SCID pigs with all genotypes. Pigs shown include a carrier (p18), *ART16/16* (p19, p20, p21, and p17), *ART12/16* (p22, p23, p24), and *ART12/12* (p25 and p6). Bands with asterisks were sequenced to confirm presence of V gene segment within the amplificon (Figures S2, S3).

Artemis is involved in DNA repair and is a critical component of VDJ recombination. Since we observed that CD3ε^+^ cells develop in *ART*^−/−^ SCID pigs, we assessed if there was sufficient residual Artemis function for productive somatic recombination by performing genomic PCR analysis on the TCRδ and TCRβ loci. PCR analysis was performed using primers that were specific for TCRδV5 and TCRδJ1, as well as TCRβV20 and TCRβJ1.2 in an assay originally described by Suzuki et al. (18) (Figure 5). We chose these primer pairs because they had previously been used to assess for VDJ recombination in a colony of *RAG2* knock out pigs (18). Due to variation within V gene segments, amplification of specific recombined gene fragments can be difficult, however the previously mentioned primer sets were confirmed to amplify PCR fragments that specifically contained the designated V and J gene segments.

SCID pigs of all three genotypes (p6, p16-25) were assessed. At least one pig from each SCID genotype had recombination between the V and J segments for either TCR complexes (TCRβ or TCRδ), although not every animal showed evidence of recombination. PCR was performed on a wild type, *ART16/*+ pig (p18) as a control and we detected rearrangements for both primer sets. PCR products (with asterisks) were sequenced to confirm amplicon contained V and J gene segments (Supplemental Figures 2, 3). In our control wildtype amplificons, we were unable to sequence through the VDJ joint, which we hypothesized was due to variable sequence joints using those specific V and J gene segments within wildtype animals. However, two SCID pigs (p20 and p24) had amplicons that sequenced through the TCRδ VDJ joint, indicating that the there was only a single clone for the V and J gene segments tested (Supplemental Figure 4). Together, we show VDJ recombination can occur in SCID pigs, although the repertoire may be limited.

### TCR Transcripts Are Expressed in SCID Thymic and Lymph Node Tissue

**Figure 6:**
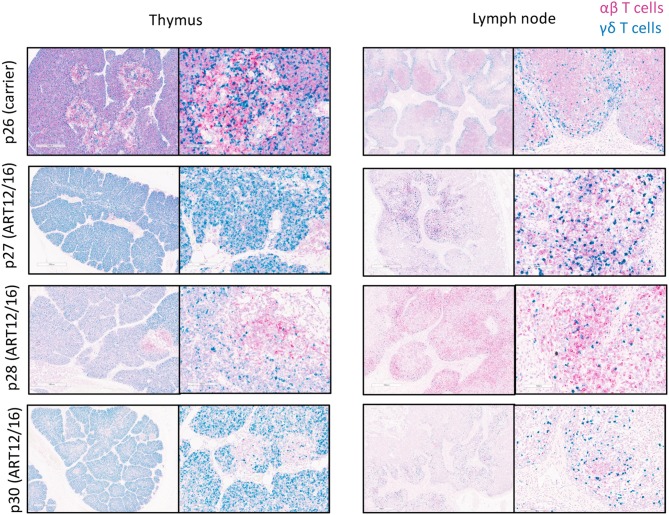
αβ and γδ T cells are in SCID pig thymic tissue and lymph nodes. Lymph nodes and remnant thymic tissue were collected from a carrier (p26) and *ART12/16* SCID pigs (p27, p28, and p30). Probes were created to target the constant region of TCRα (red) and TCRδ (green) transcripts, to detect αβ and γδ T cells, respectively. All three *ART12/16* SCID pig have both αβ and γδ T cells within the thymic and lymph node tissue, however the distribution of the cells and lymphoid organization differs from carrier animals.

Because we could detect productive somatic recombination products at the TCR loci, we wanted to determine if TCR transcripts were being expressed within SCID pig lymphoid tissues. Probes were created to target swine TRAC (pink) and TRDC (blue) transcripts and were used for the detection in thymic and lymph node tissue from one carrier (p26) and four (*ART12/16*) SCID pigs (p27-p30) (Figure 6). The carrier animal, p26, had a defined cortex and medullary areas within the thymus. *ART12/16* SCID pigs lacked this architecture, although some structure was present within the thymus from SCID p30. Additionally, in the carrier lymph node there are defined areas of αβ and γδ T cells, with γδ localized near the connective tissue and αβ cells in the interior of the lymph node nodules. SCID pig lymph nodes had TRAC and TRAD expressing T cells near the connective tissue, however the localization of these cells was less organized compared to the carrier pig. All four *ART12/16* SCID pig thymi and lymph node tissues expressed TCRα and TCRδ transcripts. Additionally, there appears to be greater abundance of TRDC transcripts in the SCID pigs compared to the carrier animal.

## Discussion

Here we report the description of T cells in SCID pigs with naturally occurring mutations in *ART*, a phenotype referred to as leaky SCID. We first observed that CD3ε^+^ cells were capable of developing in these SCID pigs when two *ART16/16* bone marrow transplanted pigs developed host-derived T cell lymphoma (3). We followed these findings by asking if CD3ε^+^ cells were found in circulation of SCID pigs housed in conventional settings and biocontainment. We found that SCID pigs raised in either setting and across all three SCID genotypes (*ART16/16, ART12/16*, and *ART12/12*) had a leaky T cell phenotype. We next assessed if newborn SCID pigs had CD3ε^+^ cells in lymph nodes to gain insight into when these cells could be developing. We found CD3ε^+^ cells in lymph nodes of all SCID genotypes, although *ART12/12* SCID pigs had fewer cells in pigs that we analyzed. We further assessed this phenotype and assayed for VDJ recombination by amplifying TCRβ and δ with V and J specific primers and found that all three SCID genotypes are capable of VDJ recombination. We suspect the TCR repertoire is limited in these SCIDs, as not all SCID pigs had recombination, and in some animals, there appears to be a clonal repertoire for the recombination events tested (Supplemental Figure 4). Lastly, we confirmed that TCRα and TCRδ transcripts are expressed in the thymic and lymph node tissues of SCID pigs.

Hypomorphic mutations are reported in human patients within *RAG1* (5, 6), *ARTEMIS* (7–10), and *IL2RG* (22) genes. Such mutations can lead to Omenn's syndrome (11) or cancer development (7, 9). Omenn's syndrome is characterized by the development and expansion of a population of self-reactive T cells. Symptoms of Omenn's syndrome include diarrhea, eosinophilia, and lethargy (23). Additionally, T-cell lymphomas can develop due to recombination errors made during VDJ recombination (8). Identifying that SCID pigs with *ART16* and *ART12* alleles can produce CD3ε^+^ cells suggests that these types of complications could arise in SCID pigs. We have previously had SCID pigs with severe rashes, diarrhea, as well as masses of lymphoid tissue that may be explained as Omenn's syndrome or cancer (unpublished observations). The characterization of CD3ε^+^ cell development in our SCID pigs suggest that these ailments could occur in our *ART* SCID model. Additional investigation into the function of the *ART16* and *ART12* alleles and potential protein products in the development of CD3ε^+^ cells will also be important as this model is developed.

Further investigation into CD3ε^+^ cell function and cytokine production will be required for better understanding of these cells within the SCID pig. Additionally, assessment of the TCRα and TCRγ recombination events would help to better characterize these animals. RNA sequencing of the repertoire of these animals would be useful in gaining a better understanding about the development of these cells (24). We have recently generated an *ART*^−/−^ *IL2RG*^−/γ^ SCID pig model, in which we show that these “leaky” CD3ε^+^ cells are not present in piglets, as these animals do not have any CD3ε^+^ cells in blood or lymphoid organs (25). Researchers and veterinarians working with SCID pigs need to be aware of the potential of leaky T cell development. Presence of these cells could affect the health and potential use of these animals in different biomedical models.

## Data Availability Statement

The raw data supporting the conclusions of this article will be made available by the authors, without undue reservation, to any qualified researcher.

## Ethics Statement

The animal study was reviewed and approved by the Iowa State University Institutional Animal Care and Use Committee.

## Author Contributions

AB designed experiments, ran flow cytometry, and wrote manuscript. AC-O and JO performed immunohistochemistry on tissues. YS and SAC performed TCR PCR analysis on lymph node DNA. JW performed RNA *in-situ* hybridization on lymphoid tissues. EP performed flow cytometry experiments. AA performed necropsies on animals and collected tissue and blood samples. CL and JC were involved in experimental planning and immunological data analysis. RR and JD were involved in experiment planning. SEC was involved with SCID pig care, genotyping, and tissue collection. CT was involved in all aspects of design and interpretation of the research. All authors read and edited the manuscript.

### Conflict of Interest

The authors declare that the research was conducted in the absence of any commercial or financial relationships that could be construed as a potential conflict of interest.

## Abbreviations

SCID: severe combined immunodeficient
TCR: T cell receptor
VDJ: variable; diversity; and joining
BMT: bone marrow transplant
PBMC: peripheral blood mononuclear cells
