# Supplementary material for: CD3ε+ Cells in Pigs With Severe Combined Immunodeficiency Due to Defects in ARTEMIS
Source: Front Immunol. 2020 Mar 31;11:510. doi: 10.3389/fimmu.2020.00510 (PMC7136459; doi:10.3389/fimmu.2020.00510)
Supplement: Supplementary file 1 [file Data_Sheet_1.docx]

**Supplemental Figures**

**
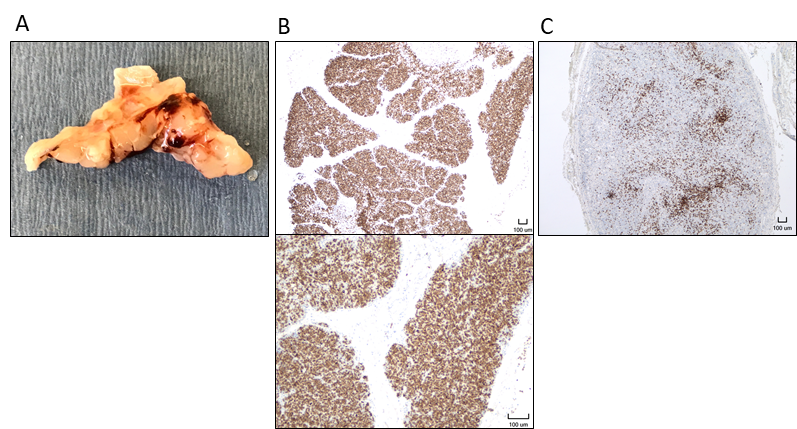
**

**Supplemental Figure 1. Thymic and lymph node tissue from an *Art16/16* SCID pig (p4) stained positively for CD3ε**

**(A)** P4 (*Art16/16*) had thymic tissue present at 4 months of age. **(B)** Thymic tissue and **(C)** lymph node tissue were stained for CD3ε. **(B)** Thymic tissue stained robustly positive for CD3ε. **(C)** CD3ε^+^ cells were present within the lymph node as well. Interestingly, this animal did not have remarkable levels of circulating CD3ε^+^ cells **(Figure 2).**

**TCR_DeltaV5** **CTCAGAGATTTAAGGAAACTGATTTTATTCTATATCCTCCACCTGAAAGGAAAAGAAAATGATTCTTGTGGGCTTTGGCC**

Pig18_Carrier **CTCAGAGATTTAAGGAAACTGATTTTATTCTATATCCTCCACCTGAAAGGAAAAGAAAATGATTCTTGTGGGCTTTGGCC**

Pig20_1616 **CT**ATN**AGATTT**G**A**A**GAAACTGATTTTATTCTATATCCTCCACCTGAAAGGAAAAGAAAATGATTCTTGTGGGCTTTGGCC**

Pig21_1616 **C**CA**A**C**A**TT**TTT**CCT**GA**TCT**T**CCC**TT**ATTCTGTCTC**C**TCCAC**C**TG**GAAA**A**GAAAAGAAAATGATTC**C**T**C**T**GCAC**T**C**T**CC**CC**

Pig23_1216 NCATT**A**TG**TTT**GT**GGAA**CA**T**TTC**TTTATTCT**C**TA**C**CCTCCACCTGAAA**T**GAAAAGAAA**C**TGATTCTTGT**GT**GCTTT**AG**CC**

Pig24_1216 **CTC**A**GAGATTTAAGGAAACTGATTTTATTCTATATCC**T**CCACCTGAAAGGAAAAGAAAATGATTCTTGTGGGCTTTGGCC**

Pig6_1212 AAGT**G**G**G**CCTATT**G**CCTGNACTGACTT**TT**T**T**C**T**CAAAC**CC**C**CCTGAA**T**GGAAAA**A**AAAATGATTCTTGTGGGCTT**NAN**CC**

**TCR_DeltaJ1** --------------------------------------------------------------------------------

**TCR_DeltaV5** **TTTTGTTTTTCTGTAAGTACATTTGTTCTCACGTCCCACTTGTTCTACCGCCCTGATTCCCTTTTTCTGACTTTCTTAAG**

Pig18_Carrier **TTTTGTTTTTCTGTAAGTACATTTGTTCTCACGTCCCACTTGTTCTACCGCCCTGATTCCCTTTTTCTGACTTTCTTAAG**

Pig20_1616 **TTTTGTTTTTCTGTAAGTACATTTGTTCTCACGTCCCACTTGTTCTACCGCCCTGATTCCCTTTTTCTGACTTTCTTAAG**

Pig21_1616 A**T**G**T**C**T**G**TTTCT**C**TA**CT**T**G**CATTTGTTC**A**CACGTCCCACTTGTTCTACCGCCCTGATTCCCTTTTTCTGACTTTCTTAA**C

Pig23_1216 **TTTT**G**TTTTTCTGTAAGTACATTTGTTCTCACGTCCCACTTGTTCTACCGCCCTGATTCCCTTTTTCTGACTTTCTTAAG**

Pig24_1216 **TTTT**G**TTTTTCTGTAAGTACATTTGTTCTCACGTCCCACTTGTTCTACCGCCCTGATTCCCTTTTTCTGACTTTCTTAAG**

Pig6_1212 **TTTT**G**TTTTTCTGTAAGTACATTTGTTCTCACGTCCCACTTGTTCTACCGCCCTGATTCCCTTTTTCTGACTTTCTTAAG**

**TCR_DeltaJ1** --------------------------------------------------------------------------------

**TCR_DeltaV5** **GCTTGGTTTCTTTTTCATTCACTTGGCTTTGCAGTGATTTTCTGCCTTTATTTTTCATCTGTGTGTCCCTCCCCTGCCTC**

Pig18_Carrier **GCTTGGTTTCTTTTTCATTCACTTGGCTTTGCAGTGATTTTCTGCCTTTATTTTTCATCTGTGTGTCCCTCCCCTGCCTC**

Pig20_1616 **GCTTGGTTTCTTTTTCATTCACTTGGCTTTGCAGTGATTTTCTGCCTTTATTTTTCATCTGTGTGTCCCTCCCCTGCCTC**

Pig21_1616 **GCTTGGTTTCTTTTTCATTCACTTGGCTTTGCAGTGATTTTCTGCCTTTATTTTTCATCTGTGTGTCCCTCCCCTGCCTC**

Pig23_1216 **GCTTGGTTTCTTTTTCATTCACTTGGCTTTGCAGTGATTTTCTGCCTTTATTTTTCATCTGTGTGTCCCTCCCCTGCCTC**

Pig24_1216 **GCTTGGTTTCTTTTTCATTCACTTGGCTTTGCAGTGATTTTCTGCCTTTATTTTTCATCTGTGTGTCCCTCCCCTGCCTC**

Pig6_1212 **GCTTGGTTTCTTTTTCATTCACTTGGCTTTGCAGTGATTTTCTGCCTTTATTTTTCATCTGTGTGTCCCTCCCCTGCCTC**

**TCR_DeltaJ1** --------------------------------------------------------------------------------

**TCR_DeltaV5** **TGCCCATTGAGTTCCCTCAGGGTGGGCTTGTGCTGGACCCCGTGCCTGTGGAGTGCAGGACTTATACCTTCCTTATTTCC**

Pig18_Carrier **TGCCCATTGAGTTCCCTCAGGGTGGGCTTGTGCTGGACCCCGTGCCTGTGGAGTGCAGGACTTATACCTTCCTTATTTCC** Pig20_1616 **TGCCCATTGAGTTCCCTCAGGGTGGGCTTGTGCTGGACCCCGTGCCTGTGGAGTGCAGGACTTAT**GT**CTTCCTTATTTCC**

Pig21_1616 **TGCCCATTGAGTTCCCTCAGGGTGGGCTTGTGCTGGACCCCG**C**GCCTGTGGAGTGCAGGACTTATACCTTCCTTATTTCC**

Pig23_1216 **TGCCCATTGAGTTCCCTCAGGGTGGGCTTGTGCTGGACCCCGTGCCTGTGGAGTGCAGGACTTATACCTTCCTTATTTCC**

Pig24_1216 **TGCCCATTGAGTTCCCTCAGGGTGGGCTTGTGCTGGACCCCGTGCCTGTGGAGTGCAGGACTTATACCTTCCTTATTTCC**

Pig6_1212 **TGCCCATTGAGTTCCCTCAGGGTGGGCTTGTGCTGGACCCCGTGCCTGTGGAGTGCAGGACTTATACCTTCCTTATTTCC**

**TCR_DeltaJ1** --------------------------------------------------------------------------------

**TCR_DeltaV5** **TGTTTCCTTATTTCTTCTCTTCACAGACAAGGGTGTGCTGTGTAACAAAGTGACCCAGACTTCCCTGGAAGAGGTGGTGG**

Pig18_Carrier **TGTTTCCTTATTTCTTCTCTTCACAGACAAGGGTGTGCTGTGTAACAAAGTGACCCAGACTTCCCTGGAAGAGGTGGTGG**

Pig20_1616 **TGTTTCCTTATTTCTTCTCTTCACAGACAAGGGTGTGCTGTGTAACAAAGTGACCCAGACTTCCCTGGAAGAGGTGGTGG**

Pig21_1616 **TGTTTCCTTATTTCTTCTCTTCACAGACAAGGGTGTGCTGTGTAACAAAGTGACCCAGACTTCCCTGGAAGAGGTGGTGG**

Pig23_1216 **TGTTTCCTTATTTCTTCTCTTCACAGACAAGGGTGTGCTGTGTAACAAAGTGACCCAGACTTCCCTGGAAGAGGTGGTGG**

Pig24_1216 **TGTTTCCTTATTTCTTCTCTTCACAGACAAGGGTGTGCTGTGTAACAAAGTGACCCAGACTTCCCTGGAAGAGGTGGTGG**

Pig6_1212 **TGTTTCCTTATTTCTTCTCTTCACAGACAAGGGTGTGCTGTGTAACAAAGTGACCCAGACTTCCCTGGAAGAGGTGG**A**GG**

**TCR_DeltaJ1** --------------------------------------------------------------------------------

**TCR_DeltaV5** **TGAGTGGCAGTAAGGTGACACTGCCCTGCACTTTTGAAACCTCACACTCAGATCCAGACCTCTACTGGTACCGAATACGT**

Pig18_Carrier **TGAGTGGCAGTAAGGTGACACTGCCCTGCACTTTTGAAACCTCACACTCAGATCCAGACCTCTACTGGTACCGAATACGT**

Pig20_1616 **TGAGTGGCAGTAAGGTGACACTGCCCTGCACTTTTGAAACCTCACACTCA**A**ATCCAGACCTCTACTGGTACCGAATACGT**

Pig21_1616 **TGAGTGGCAGTAA**A**GTGACACTGCCCTGCACTTTTGAAACCTCACACTCA**A**ATCCAGACCTCTACTGGTACCGAATACGT**

Pig23_1216 **TGAGTGGCAGTAAGGTGACACTGCCCTGCACTTTTGAAACCTCACACTCAGATCCAGACCTCTACTGGTACCGAATACGT**

Pig24_1216 **TGAGTGGCAGTAAGGTGACACTGCCCTGCACTTTTGAAACCTCACACTCAGATCCAGACCTCTACTGGTACCGAATACGT**

Pig6_1212 **TGAGTGGCAGTAAGGTGACACTGCCCTGCACTTTTGAAACCTCACACTCAGATCCAGACCTCTACTGG**C**ACCGAATACGT**

**TCR_DeltaJ1** ------------------------------------------------------------ --------------------

**TCR_DeltaV5** **CCAGATCGTACCTTCCAGCTTGTCTTGTACAGGGATAATACTAGGTCCTACGATGCT-GATTTTGCTCGGGGTAGATTTT**

Pig18_Carrier **CCAGATCGTACCTTCCAGCTTGTCTTGTACAGGGATAATACTAGGTCCTACGATGCT-GATTTTGCTCGGGGTAGATTTT**

Pig20_1616 **CCAGATCGTACCTTCCAG**T**TTGTCTTGTACAGGAATAATACTAGGTCCTACGATGCT-GATTTTGCTCGGGGTAGATTTT**

Pig21_1616 **CC**T**GATCGTACCTTCCAG**A**TTG**N**CTTGTACACGGAT**C**ATACTAGGTCCTACGATGCT**T**GATTTTGCTC**T**GG**T**TA**T**ATTTT**

Pig23_1216 **CCAGATCGTACCTTCCAGCTTGTCTTGTACAGGGATAATACTAGGTCCTACGATGCT**-**GATTTTGCTCGGGGTAGATTTT**

Pig24_1216 **CCAGATCGTACCTTCCAGCTTGTCTTGTACAGGGATAATACTAGGTCCTACGATGCT**-**GATTTTGCTCGGGGTAGATTTT**

Pig6_1212 **CCAGATCG**C**ACCTTCCAGCTTGTCTTGTACAGGGATAATACTAGGTCCTACGATGCT**-**GATTTTGCTCGGGGTAGATTTT**

**TCR_DeltaJ1** --------------------------------------------------------------------------------

**TCR_DeltaV5** **C-TGTGCAGCACAGTCTGGCCCACAAAACCTTTCACTTGGTGATCTCCTCAGTGACAACTAAAGACACTGCCACTTACT**

Pig18_Carrier **C-TGTGCAGCACAGTCTGGCCCACAAAACCTTTCACTTGGTGATCTCCTCAGTGACAACTAAAGACACTGCCACTTACT**

Pig20_1616 **C-TGTGCAGCACAGTCTGGCCCACAAAACCTTTCACTTGGTGATCTCCTCAGTGACAACTAAAGACACTGCCACTTACT**

Pig21_1616 **C**C**TGTGC**TT**CACAGTCTGGCCCACAAAACCTATCACTTGGTGA**G**CTCCTCATTGACAACTAAAGACACTGCCACTTTA**C

Pig23_1216 **C-TGTGCAGCACAGTCTGGCCCACAAAACCTTTCACTTGGTGATCTCCTCAGTGACAACTAAAGACACTGCCACTTACT**

Pig24_1216 **C-TGTGCAGCACAGTCTGGCCCACAAAACCTTTCACTTGGTGATCTCCTCAGTGACAACTAAAGACACTGCCACTTACT**

Pig6_1212 **C-TGTGCAGCACAGTCTGGCCCACA**T**AACCTTTCACTTGGTGATCTCCTCAGTGACAACTAAAGACACTGCCACTTACT**

**TCR_DeltaJ1** ------------------------------------------------------------------------------

**TCR_DeltaV5** **ACTGTGCCTT-GGACTCC**------------------------------------------ -----------------

Pig18_Carrier **ACTGTGCCTT**-**GGACTCC**------------------------------------------ -----------------

Pig20_1616 **ACTGTGCCTT**-**GGACTC**------------CGG**AGATAAACTCATCTTTGGA**-**AAAGGGACTCAGCTGGTTGTGGAAC**

Pig21_1616 TACT**T**TGCCCTTGNNNNGTCACCTACCTTCCGTAG**TA**T**ACT**ACATC**T**ATC**A**G**AAAGGG**T**C**A**C**TCAAAC**TT**C**T**TT---

Pig23_1216 **ACTGTGCCTT**-**GGACTCC**GGAGTCG**AT**ATACTC**G-T**------------------------ -----------------

Pig24_1216 **ACTGTGCCTT**-**G**ACGAGATCTCGTACCTCCGG**AG**G**TAAACTCATCTTTGGA**-**AAAGGGACTCAGCTGGTTGTGGAAC**

Pig6_1212 **ACTGCGCCTT**-**GGA**A**TC**NGCATGTNNN**G**C**TAC**G**G**N**T**C**A**N**CT**AG**T**TG**T**N**GGA**-C**A**NNT**G**CG**TCA**ATA**G**CC**T**C**TG**C**AAC**

**TCR_DeltaJ1** -------------------------**ATGATACAGATAAACTCATCTTTGGA**-**AAAGGG**AC**TCAGCTGGTTGTGGAAC**

**Supplemental Figure 2. Sequence alignment of TCRδ V5 and J1 products from carrier and SCID pigs.**

DNA from lymph node tissue was amplified with primers specific for TCRδV5 and TCRδ J1. DNA products from a carrier and SCID animals were sequenced and then aligned with TCRδ V5 and TCRδ J1 (derived from accession number AB182371.1 from NCBI). Red indicate the TCRδ V5 sequence and blue indicate the TCRδ J1. Bold nucleotides indicate nucleotides that match the reference sequence.

**TCR_BetaV20**  **GTCTGATCAAAACAAGAGGGGACCTCCACGCTTTCTGGAGGTGGAAAGGGTAAAGCGAGAAGCCACCAGTCGTGGGGGGA**

Pig18_Carrier **G**G**C**G**GATCAAAACAAGAGGGGACCTCCACGCT**C**TCTGGAGGTGGAAAGGGTAAAGCGAGAAGCCACCAGTCG**C**GGGGG**A-

Pig20_1616 **G**G**CTGATCAAAACAAGAGGGGACCTCCACGCT**C**TCTGGAGGTGGAAAGGGTAAAGCGAGAAGCCACCAGTCGCGGGGG**A-

Pig21_1616 **GTCTGATCAAAACAAGAGGGGACCTCCACGCTTTCTGGAGGTGGAAAGGGTAAAGCGAGAAGCCACC**T**GTCGTGGGGG**A-

Pig22_1216 **G**G**CTGATCAAAACAAGAGGGGACCTCC**T**CGCT**C**TCTGGAGGTGGAAAGGGTAAAGCGAGAAGCCACC**T**GTCGTGGGGG**A-

Pig24_1216 **G**G**CTGATCAAAACAAGAGGGGACCTCC**T**CGCT**C**TCTGGAGGTGGAAAGGGTAAAGCGAGAAGCCACC**T**GTCGTGGGGG**T-

**TCR_BetaJ1.1**  --------------------------------------------------------------------------------

**TCR_BetaV20**  **GTGAGGGTTATG-ACCCTAAGAAGTAGGACAGAGAGACTTCGGAAAGCCAATGGGATTCCCTGGGAGCTGTGAGAAAGGG**

Pig18_Carrier **GTGAGGGTTATG-ACCCTAAGAAGT**G**GGACAGAGAGACTTCGGAAAGCCA**G**TGGGATTCCCTGGGAGCTGTGAGAAAGGG**

Pig20_1616 **GTGAGGGTTATG-ACCCTAAGAAGT**G**GGACAGAGAGACTTCGGAAAGCCA**G**TGGGATTCCCTGGGAGCTGTGAGAAAGGG**

Pig21_1616 **GTGAGGGTTATG-ACCCTAAGAAGTAGGACAGAGAGACTTCGGAAAGCCAATGGGATTCCCTGGGAGCTGTGAGAAAGGG**

Pig22_1216 **GTGAGGGTTATG**G**ACCCTAAGAAGTAGGACAG**T**GAGACTTC**A**GAAAGCCAATGGGATTCCCTGGGAGCTGTGAGAAAGGG**

Pig24_1216 **GTGAGGGTTATG**G**ACCCTAAGAAGTAGGACAG**T**GAGACTTC**A**GAAAGCCAATGGGATTCCCTGGGAGCTGTGAGAAAGGG**

**TCR_BetaJ1.1**  ---------------------------------------------------------------------------------

**TCR_BetaV20**  **AAGGGAGCCAGGCTGATTTTCTGCCCATGGTCAGGTCCGCCTGGTCCTGCCGGCTGCTCATCTCTTTGACCTCTGTCTCA**

Pig18_Carrier **AAGGGAGCCAGGCTGATTTTCTGCCCATGGTCAGGTCCGCCTGGTCCTGCCGGCTGCTCATCTCTTTGACCTCTGTCTCA**

Pig20_1616 **AAGGGAGCCAGGCTGATTTTCTGCCCATGGTCAGGTCCGCCTGGTCCTGCCGGCTGCTCATCTCTTTGACCTCTGTCTCA**

Pig21_1616 **AAGGGAGCCAGGCTGATTTTCTGCCCATGGTCAGGTCCGCCTGGTCCTGCCGGCTGCTCATCTCTTTGACCTCTGTCTCA**

Pig22_1216 **AAGGGAGCCAGGCTGATTTTCTGCCCATGGTCGGGTCCGCCTGGTCCTGCCGGCTGCTCATCTCTTTGACCTCTGTCTCA**

Pig24_1216 **AAGGGAGCCAGGCTGATTTTCTGCCCATGGTCGGGTCCGCCTGGTCCTGCCGGCTGCTCATCTCTTTGACCTCTGTCTCA**

**TCR_BetaJ1.1**  --------------------------------------------------------------------------------

**TCR_BetaV20**  **GCAGGCTACGGGTTTGGCGCCCTCGTCTCTCAACATCCCGGCAGGGCCATCTGTAAGAGTGGTGCCTCTGTGACCATCCA**

Pig18_Carrier **GCAGGCTACGGGTTTGGCGCCCTCGTCTCTCAACATCCC**A**GCAGGGCCATCTGTAAGAGCGGTGCCTCTGTGACCATCCA**

Pig20_1616 **GCAGGCTACGGGTTTGGCGCCCTCGTCTCTCAACATCCC**A**GCAGGGCCATCTGTAAGAGCGGTGCCTCTGTGACCATCCA**

Pig21_1616 **GCAGGCTACGGGTTTGGCGCCCTCGTCTCTCAACATCCCGGCAGGGCCATCTGTAAGAGTGGTGCCTCTGTGACCATCCA**

Pig22_1216 **GCAGGCTACGGGTTTGGCGCCCTCGTCTCTCAACATCCCGGCAGGGCCATCTGTAAGAGCGGTGCCTCTGTGACCATCCA**

Pig24_1216 **GCAGGCTACGGGTTTGGCGCCCTCGTCTCTCAACATCCCGGCAGGGCCATCTGTAAGAGCGGTGCCTCTGTGACCATCCA**

**TCR_BetaJ1.1**  --------------------------------------------------------------------------------

**TCR_BetaV20**  **GTGCCGTACAGTGGACCTTCAAGCCACAACTATGTTCTGGTATCATCAGTTCCCAGAACAGGGCCCCCTGCTGATAGCAA**

Pig18_Carrier **GTGCCGTACAGTGGAC**T**TTCAA**A**CCACAACTATGTTCTGGTATCATCAGTTCCCAGAACAGGGCCCCCTGCTGATAGCAA**

Pig20_1616 **GTGCCGTACAGTGGACTTTCAA**A**CCACAACTATGTTCTGGTATCATCAGTTCCCAGAACAGGGCCCCCTGCTGATAGCAA**

Pig21_1616 **GTGCCGTACAGTGGACCTTCAAGCCACAACTATGTTCTGGTATCATCAGTTCCCAGAACAGGGCCCCCTGCTGATAGCAA**

Pig22_1216 **GTGCCGTACAGTGGACCTTCAAGCCATAACTATGTTCTGGTATCATCAGTTCCCAGAACAGGGCCCCCTGCTGATAGCAA**

Pig24_1216 **GTGCCGTACAGTGGACCTTCAAGCCATAACTATGTTCTGGTATCATCAGTTCCCAGAACAGGGCCCCCTGCTGATAGCAA**

**TCR_BetaJ1.1**  --------------------------------------------------------------------------------

**TCR_BetaV20**  **CTTCTAACATGGGCTCTAATGCCACCTACGAAAAAGGTTATAACAGCGCCAAGTTTCTCATCAGCCACCCAAACCAAAGG**

Pig18_Carrier **CTTCTAACATGGGCTCTAATGCCACCTACGAAAAAGGTTTTAACAGC**A**CCAAGTTTCTCATCAGCCACCCA**GA**CC**T**AA**C**G**

Pig20_1616 **CTTCTAACATGGGCTCTAATGCCACCTACGAAAAAGGTTTTAACAGC**A**CCAAGTTTCTCATCAGCCACCCA**G**ACC**T**AACG**

Pig21_1616 **CTTCTAACATGGGCTCTAATGCCACCTACGAAAAAGGTTATAACAGCGCCAAGTTTCTCATCAGCCACCCAAACCAAAGG**

Pig22_1216 **CTTCT**A**ACATGGGCTCTAATGCCACCTACGAAAAAGGTTATAACAGCGCCAAGTTTCTCATCAGCCACCCA**G**ACCAAA**C**G**

Pig24_1216 **CTTCT**G**AC**G**TGGGCTCTAATGCCACCTACGAAAAAGGTT**A**TAACAGCGCCAAGTTTCTCATCAGCCACCCA**G**ACCAAA**C**G**

**TCR_BetaJ1.1**  --------------------------------------------------------------------------------

**TCR_BetaV20**  **TTTTCATCTCTGGTGGTGAGAAGCGTGCATCCTGCCGACAGCAGCCTCTAC-TTTTGTGGTGCTAGTGA**-----------

Pig18_Carrier **TTTTCATCTCTGGTGGTGAGACGCGTGCATCCTGCCGACAGCAGCCTCTAC**C**TTT-GTGGTGCTA**GCCTCAGGACGGGGA

Pig20_1616 **TTTTCATCTCTGGTGGTGAGACGCGTGCATCCTGCCGACAGCAGCCTCTAC**C**TTT**-**GTG**GTACACACGGGGCAGG**A**TT**CA**

Pig21_1616 **TTTTCATCTCTGGTGGTGAGAAGCGTGCATCCTGCCGACAGCAGCCTCTAC**-**TTT**-**GTGG**CTCCCCGNCCC---------

Pig22_1216 **TTTTCATCTCTGGTGGTGAGAAGCGTGCATCCTGCCGACAGCAGCCTCTACCTTT**-**G**GCTGCNNNCCCTCGCCCCC----

Pig24_1216 **TTTTCATCTCTGGTGGTGAGAAGCGTGCATCCTGCCGACAGCAGCCTCTACCTT**--**GT**TAGAGAGCCCCCC---------

**TCR_BetaJ1.1**  --------------------------------------------------------------------------**CAAACA**

**TCR_BetaV20**  -------------------------------------------

Pig18_Carrier AC-**G**NN**GTCTTCTT**CGT**AG**AAGAGTGTNTGCCAAAAAAAACAA

Pig20_1616 **C**-**TGAAGTCTTCTTTGGAG**AATCATAGACNNNCATGGAGCACC

Pig21_1616 -------------------------------------------

Pig22_1216 -------------------------------------------

Pig24_1216 -------------------------------------------

**TCR_BetaJ1.1**  **CTTGAAGTCTTCTTTGGAGGAGGCACCAGGCTCACGGTTGTGG**

**Supplemental Figure 3. Sequence alignment of TCRβ V20 and J1.2 products from carrier and SCID pigs.**

DNA from lymph node tissue was amplified with primers specific for TCRβ V20 and TCRβ J1.2. DNA products from a carrier and SCID animals were sequenced and then aligned with TCRβV20 and TCRβJ1.1 (derived from accession number AB476299.1 from NCBI). Red indicate the TCRβV20 sequence and blue indicate TCRβJ1.1 sequence. Bold nucleotides indicate the nucleotides that match the reference sequence.

**
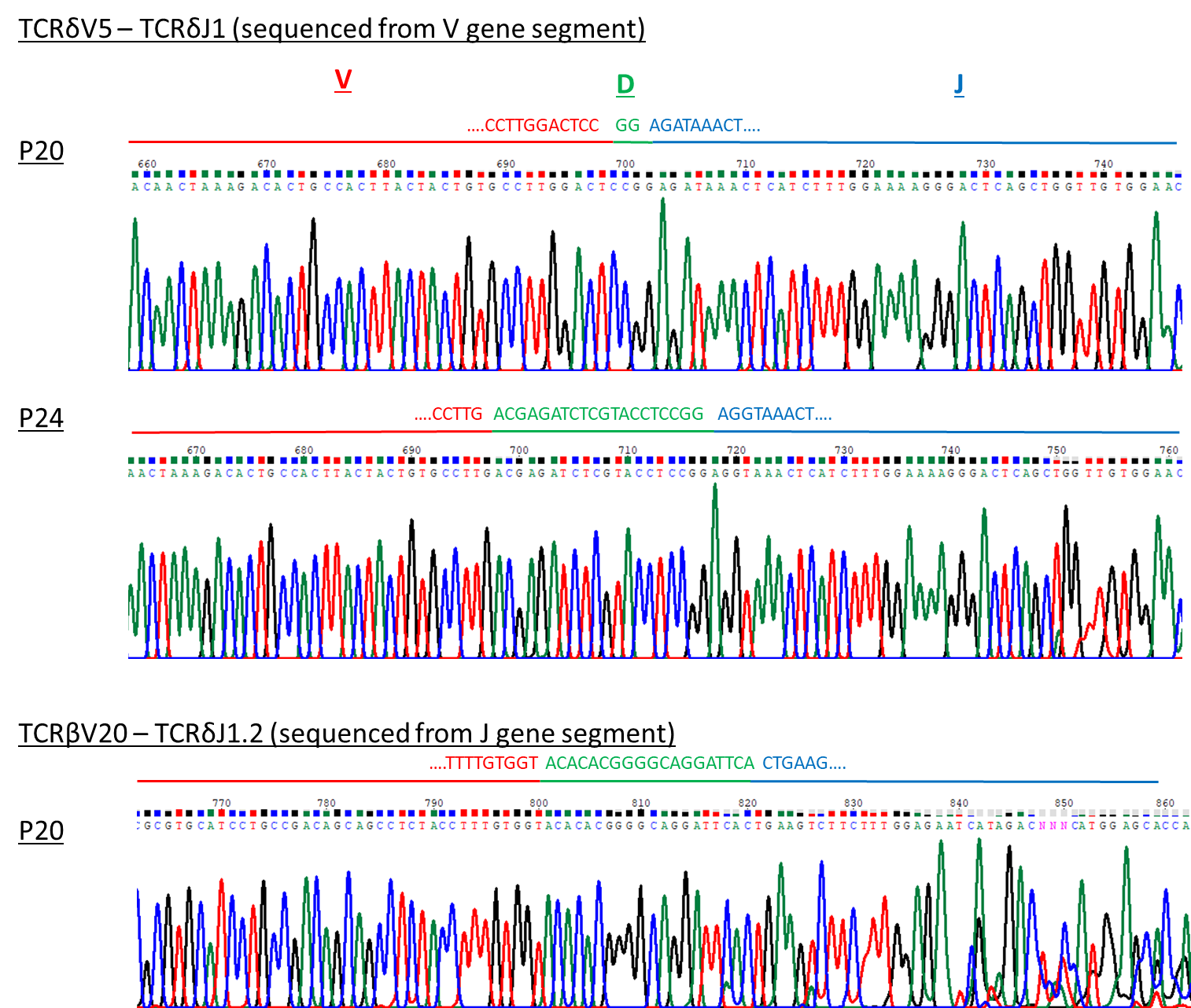
**

**Supplemental Figure 4. Chromatograms of V-D-J joins in TCRβ (V20 and J1.2) and TCRδ (V5 and J1) show evidence of single TCR clones**

Chromatograms are shown for TCRδ (top) and TCRβ (bottom) amplicons from lymph nodes of SCID pigs p20 (*Art16/16*) and p24 (*Art12/16*). Clean sequences were obtained through the VDJ joint for TCRδ and TCRβ, suggesting that the repertoire for these two rearrangements is clonal in these SCID pigs.
